# Mechanical transmission of dengue virus by *Aedes aegypti* may influence disease transmission dynamics during outbreaks

**DOI:** 10.1016/j.ebiom.2023.104723

**Published:** 2023-07-22

**Authors:** Hsing-Han Li, Matthew P. Su, Shih-Cheng Wu, Hsiao-Hui Tsou, Meng-Chun Chang, Yu-Chieh Cheng, Kuen-Nan Tsai, Hsin-Wei Wang, Guan-Hua Chen, Cheng-Kang Tang, Pei-Jung Chung, Wan-Ting Tsai, Li-Rung Huang, Yueh Andrew Yueh, Hsin-Wei Chen, Chao-Ying Pan, Omar S. Akbari, Hsiao-Han Chang, Guann-Yi Yu, John M. Marshall, Chun-Hong Chen

**Affiliations:** a National Mosquito-Borne Disease Control Research Center, NHRI, Miaoli, 350401, Taiwan; b National Institute of Infectious Diseases and Vaccinology, NHRI, Miaoli, 350401, Taiwan; c Division of Biological Sciences, Section of Cell and Developmental Biology, University of California, San Diego, La Jolla, CA, 92093, USA; d Graduate School of Science, Nagoya University, Nagoya, 464-8602, Japan; e Institute for Advanced Research, Nagoya University, Nagoya, 464-8601, Japan; f Department of Clinical Laboratory Sciences and Medical Biotechnology, College of Medicine, National Taiwan University, Taipei, 10048, Taiwan; g Department of Laboratory Medicine, National Taiwan University Hospital, College of Medicine, National Taiwan University, Taipei, 10021, Taiwan; h Institute of Population Health Sciences, National Health Research Institutes, Zhunan, Miaoli, 350401, Taiwan; i Graduate Institute of Biostatistics, College of Public Health, China Medical University, Taichung, 40402, Taiwan; j Department of Life Science & Institute of Bioinformatics and Structural Biology, National Tsing Hua University, Hsinchu, Taiwan; k Department of Biological Science and Technology, National Yang Ming Chiao Tung University, Hsinchu, 300, Taiwan; l Program of Plant Protection and Health, Academy of Circular Economy, National Chung Hsing University, Taichung, 402202, Taiwan; m Institute of Molecular and Genomic Medicine, NHRI, Miaoli, 350401, Taiwan; n Institute of Biotechnology and Pharmaceutical Research, NHRI, Miaoli, 350401, Taiwan; o Department of Health, Kaohsiung City Government, Kaohsiung, 800852, Taiwan; p Divisions of Biostatistics and Epidemiology, School of Public Health, University of California, Berkeley, CA, 94720, USA

**Keywords:** *Aedes aegypti* mosquito, Dengue transmission, Mathematical modelling of disease outbreak, Animal models of dengue virus

## Abstract

**Background:**

Dengue virus outbreaks are increasing in number and severity worldwide. Viral transmission is assumed to require a minimum time period of viral replication within the mosquito midgut. It is unknown if alternative transmission periods not requiring replication are possible.

**Methods:**

We used a mouse model of dengue virus transmission to investigate the potential of mechanical transmission of dengue virus. We investigated minimal viral titres necessary for development of symptoms in bitten mice and used resulting parameters to inform a new model of dengue virus transmission within a susceptible population.

**Findings:**

Naïve mice bitten by mosquitoes immediately after they took partial blood meals from dengue infected mice showed symptoms of dengue virus, followed by mortality. Incorporation of mechanical transmission into mathematical models of dengue virus transmission suggest that this supplemental transmission route could result in larger outbreaks which peak sooner.

**Interpretation:**

The potential of dengue transmission routes independent of midgut viral replication has implications for vector control strategies that target mosquito lifespan and suggest the possibility of similar mechanical transmission routes in other disease-carrying mosquitoes.

**Funding:**

This study was funded by grants from the National Health Research Institutes, Taiwan (04D2-MMMOST02), the 10.13039/100004412Human Frontier Science Program (RGP0033/2021), the 10.13039/100000002National Institutes of Health (1R01AI143698-01A1, R01AI151004 and DP2AI152071) and the 10.13039/501100004663Ministry of Science and Technology, Taiwan (MOST104-2321-B-400-016).

**Research in context**

**Evidence before this study**

Immediate transmission of multiple viruses, but not dengue virus, by *Aedes* mosquitoes without an extrinsic incubation period has been demonstrated. It remains unclear if dengue can be transmitted mechanically and the potential impact this transmission route could have on disease outbreak dynamics.

**Added value of this study**

Improved mouse models of dengue virus transmission allow for detailed testing of disease transmission. We utilise these models to investigate if mechanical transmission of dengue virus is possible.

**Implications of all the available evidence**

Our demonstration of mechanical transmission of dengue virus in mouse disease models implicates mechanical transmission as potentially influencing disease dynamics during crucial early outbreak stages.

## Introduction

Global transmission of dengue virus (DENV) has become a significant public health concern over the past 40 years.^1^ Almost half of the world's population is now at risk of infection,^2^ with over 390 million cases reported each year.^3^ Recent years have seen large and rapid outbreaks of dengue occurring in multiple East Asian countries,^4^, ^5^, ^6^ placing severe strain on local medical systems. This is in part due to the limited treatment options available, with DENV infection potentially causing a range of symptoms varying from mild flu-like features to severe dengue haemorrhagic fever and dengue shock syndrome.^7^

Given the paucity of treatment options, disease control heavily relies on interventions targeting the vectors themselves (*Aedes aegypti* [*Ae. aegypti*] and *Aedes albopictus*).^1^ However, DENV outbreaks can still occur within extremely short time periods with even relatively small infectious mosquito populations in dengue-receptive areas.^8^ Although there is some evidence that an elevated density of mosquitoes plays a contributing role,^5^^,^^9^ a much-improved understanding of DENV transmission routes is necessary to identify methods to slow these outbreaks.

Multiple possible viral transmission routes exist dependent on specific pathogen features, including airborne transmission of respiratory viruses,^10^ fluid exchange transmission of human immunodeficiency virus (HIV),^11^ foodborne transmission of noroviruses,^12^ and vector borne transmission of flaviviruses.^13^ These transmission routes greatly affect the infected population size during an epidemic. DENV transmission is assumed to follow a human-mosquito-human cycle.^7^ Here, mosquitoes become infected via a blood meal from an infected human, with DENV replicating in the mosquito midgut for 3–5 days before needing up to 14 days to move to the salivary gland. The mature virus is then able to infect susceptible humans via a bite from this now-infected mosquito.^7^

This time period between the mosquito becoming infected and it becoming infectious, denoted as the extrinsic incubation period (EIP), has thus been estimated at approximately 5–14 days.^14^ The EIP influences the proportion of mosquitoes that survive to become infectious after exposure to DENV, meaning it plays a key role in predicting the magnitude of dengue outbreaks.^14^, ^15^, ^16^ This property makes it useful for modelling dengue transmission dynamics,^17^^,^^18^ though parameter estimates for the EIP are highly variable.^19^

Regardless of the exact EIP value used in specific models, the assumption of a minimum necessary time period before mosquitoes become infectious is widespread; it is presumed that for time values below this EIP, DENV transmission is not possible. Thus, whilst these models are extremely useful, they are unable to account for potential alternative DENV transmission methods that do not require extrinsic incubation of the virus. These alternative transmission methods are necessary to explain spatial and temporal studies which indicate many dengue clusters occur in the same household with infection times of less than 7 days, far below the lowest possible EIP estimates. Indeed, DENV clusters (defined as two or more cases within 14 days within 150 m of each other) are highly focal in space and time^20^^,^^21^ and have even been observed within the same household.^22^

Interestingly, mechanical transmission (MT) of some viruses without an incubation period has been demonstrated in *Ae. aegypti*, including Chikungunya (CHIKV), poxviruses, myxoma virus, and lumpy skin disease virus, between animals such as rabbits, sheep, and goats.^23^, ^24^, ^25^ Though a recent study which identified viral CHIKV and DENV on the proboscises of female *Ae. aegypti* immediately after uptake of an infectious blood meal found no evidence of MT playing a role in both CHIKV and DENV transmission,^26^ this stands in direct contradiction to earlier studies that CHIKV can be mechanically transmitted by *Ae. aegypti*,^25^ may potentially be the result of the mouse strain tested.^25^^,^^26^

**Fig. 1:**
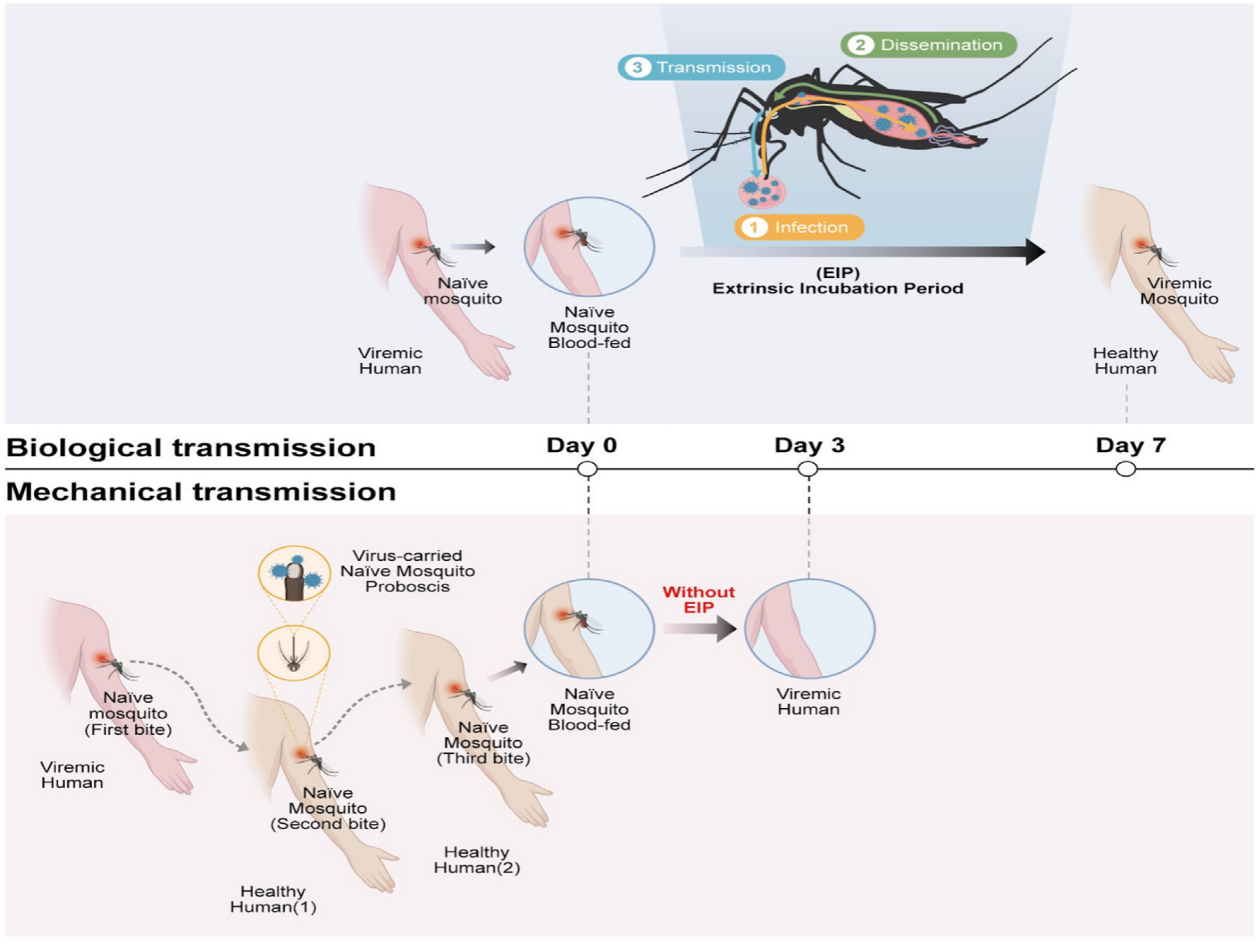
**Mechanical transmission of DENV may alter disease outbreak dynamics and influence the time between infections.** Diagrams of biological (upper panel) and mechanical (bottom panel) DENV transmission routes, with simulated data shown to the right demonstrating resulting changes in the speed and extent of disease spread.

Mechanical transmission of DENV might therefore also be possible, as *Aedes* mosquitoes may require up to four bites (potentially from multiple humans) to take a complete blood meal.^27^^,^^28^ Though it is thus far unclear if/how MT contributes to dengue outbreaks, it could act to supplement biological transmission routes of infection, boosting the number of infected individuals in the early stages of outbreaks (Fig. 1). Though there are a number of factors which could contribute to this clustering of cases within time windows less than the minimum EIP estimate, MT may act as one component.^24^^,^^29^

To explore the potential importance of mechanical transmission (MT) as a route of DENV transmission, we here conducted a mixture of simulations and laboratory experiments. Our simulations incorporating MT as a potential transmission route were similar to real-world data collected from Kaohsiung city. In our needle-sharing experiment, we found that needles puncturing DENV-infected mice could transmit the virus to naive mice and our sequential blood feeding assay demonstrated that *Ae. aegypti* mosquitoes feeding on DENV-infected mice were able to immediately transmit the virus to naive mice without an EIP. Our mathematical model incorporating parameters derived from these experiments found that MT could significantly impact the dynamics of DENV outbreaks.

Overall, our research highlights the importance of considering MT as a possible transmission route for DENV and its implications for vector control interventions. Our findings have important implications for worldwide mosquito control programs, as these programs typically focus on shortening the lifespan of *Aedes* mosquitoes, which may not be effective in preventing mechanical transmission. Our model can be used as a sentinel assay for evaluating the severity of DENV outbreaks.

## Methods

### Virus and cell maintenance

The clinical isolate 2015 DENV-2 (TW2015; GenBank: KU365901)^19^ was propagated in *Ae. aegypti* mosquitoes and Vero 76 cells (RRID: CVCL_0603). The viral titre was determined via plaque-formation assay using BHK-21 clone 13 cells (RRID: CVCL_1915). Vero cells were grown at 37 °C, 5% CO_2_ in 1 × Dulbecco's modified Eagle's medium (DMEM) supplemented with 5% foetal bovine serum (FBS), 1 × MEM nonessential amino acid solution, and 1 × antibiotic-antimycotic solution. BHK-21 clone 13 cells were grown in 1 × DMEM supplemented with 2% FBS and 1 × antibiotic-antimycotic solution at 37 °C, 5% CO_2_. All reagents were purchased from Thermo Fisher Scientific. All experiments were conducted using viral samples obtained following four passages of the original virus. All cell lines were purchased from the Food Industry Research and Development Institute, Taiwan. For mycoplasma testing: after thawing cells and culturing for at least 48 h, 1 ml of medium was centrifuged at high speed at 13,200 rpm for 10 min. The supernatant was then removed, and 20 μL of water was added for reconstitution, after which the sample was boiled at 95 °C for 10 min. Treated samples were subjected to PCR, followed by gel electrophoresis to test for the presence of mycoplasma contamination. This test was conducted approximately once per month. Mycoplasma testing was detected using a PCR with a thermal profile of step one: 94 °C for 2 min; 30 cycles of step two: 94 °C for 30 s, 60 °C for 2 min, 72 °C for 1 min; and step three: 72 °C for 5 min. The following primers were used for PCR in mycoplasma testing:

Mycoplasma forward primer: 5′-GGGAGCAAACAGGATTAGATACCCT-3’.

Mycoplasma reverse primer: 5′-TGCACCATCTGTCACTCTGTTAACCTC-3’.

### Plaque-formation assay

The viral titre of mouse serum stored at −80 °C was determined by a previously described plaque-formation assay.^30^ In brief, virus-containing serum samples from mice were serially diluted in serum-free DMEM and added to a BHK-21 clone 13 cell monolayer for virus adsorption at 37 °C for 2 h. After the diluted samples were removed, the BHK-21 clone 13 cells were overlaid with DMEM containing 1% methylcellulose (4000 cps, Sigma–Aldrich), 5% FBS, 2 mM glutamine, 1 mM sodium pyruvate, 2.5 mM HEPES, and 1 × antibiotic-antimycotic solution and cultured for 6 days at 37 °C. The overlaid medium was then gently removed, and the cells were stained with Rapid Gram Stain solution (Tonyar Biotech) for 2 h to stain plaques before being washed with water. Plaques were counted, with viral titres expressed as plaque-forming units (PFU)/mL.

### Mosquito husbandry

*Ae. aegypti* (Higgs strain; Akbari lab) mosquito eggs were hatched in deionized water under deoxygenated conditions. Hatched larvae were fed powdered yeast and goose liver (NTN Fishing Bait). Newly emerged mosquitoes were collected in rearing cages and fed a 10% sucrose solution for maintenance at 28 °C in 70% relative humidity with a 12-h light/dark cycle. Five-day-old female mosquitoes were starved for 16 h prior to mechanical DENV transmission experiments.

### Ethics

All animal experiments were conducted in compliance with the Guidelines for the Care and Use of Laboratory Animals published by the National Research Council, Taiwan (1996). The animal protocol was approved by the Institutional Animal Care and Use Committee of the National Health Research Institutes (NHRI-IACUC-107054-A). The Laboratory Animal Center at the NHRI were mouse husbandry was conducted received full accreditation from the Association for Assessment and Accreditation of Laboratory Animal Care (AAALAC) International in 2015.

### Mouse husbandry

AGB6 mice, which are deficient in both type I and II interferon signalling, used for all experiments were a cross between *Ifnar*^−/−^ mice and *Ifngr*^−/−^ mice (both with a C57BL/6 background; Jackson Laboratory, Bar Harbor, ME, USA).^19^^,^^31^ Mice were bred and maintained in the ABSL-2 animal feeding room of the Laboratory Animal Center at the NHRI at 22 ± 2 °C in 55 ± 10% relative humidity with a 12-h light/dark cycle. All food, water, aspen chips and cages were sterilized before use. Mouse health monitoring was performed every 3, 6, or 12 months by Serology, Microbiology and Parasites, using the standard operating protocol of the NHRI Animal Center (https://lac.nhri.org.tw/08-2/).

### Kaohsiung city household infection data analysis

Household level infection data was obtained from Department of Health, Kaohsiung City Government. In this dataset, the time between DENV infections reported from single households containing multiple individuals (as determined via Dengue NS1 Rapid Test (SD BIOLINE Inc., Korea; sensitivity and specificity estimated at 92 and 98%, respectively) assays) was calculated.

### Infection of mice with DENV

Eight-to ten-week-old, 18–24 g female and/or 22–25 g male AGB6 mice were challenged intravenously with either 1000 PFU of DENV^32^ in 100 μL of serum-free DMEM or with 1 × PBS (control) three days before the start of all experiments. For DENV infection via injection of mosquito proboscis extracts, female *Ae. aegypti* were first allowed to feed from DENV-infected mice until either half or fully engorged. Mosquito proboscises were collected and dissected in groups of one, four, or ten; these were then ground in 100 μL of 1 × PBS prior to inoculating naïve AGB6 mice via intravenous injection.

### Infection of AGB6 mice with DENV via mechanical transmission

Mice were intraperitoneally injected with the anaesthetics Rompun (16 mg/kg, Bayer Animal Health) and Ketalar (100 mg/kg, Pfizer). Anesthetized mice were individually placed on top of the mesh covering a mosquito cage and exposed to three to five mosquitoes that had been starved for 16 h. These mosquitoes were allowed to feed from DENV-infected AGB6 mice until they were half engorged, as confirmed via visual observation. The mice were then immediately removed and naïve AGB6 mice were placed on the top of the cage. The mosquitoes were allowed to resume feeding until fully engorged. The number of successful mosquito bites was determined by counting the number of blood-engorged mosquitoes. Mouse serum was collected by retro-orbital bleeding on days 0, 2, 4, and 6 after mosquito biting, and body weight and survival were monitored for 10 days.

Sample sizes were based on the practical experimental considerations over mice handling. No selection criteria were applied when selecting mice for testing, and no animal data was excluded from analyses. Researchers were not blinded to the infection status of the mice during experiments.

### Quantification of DENV genomic RNA

Mosquitoes were dissected either two, four or six days after biting non-infected/DENV-infected mice. Proboscis tissues were collected, and RNA was extracted using a standard Trizol-based protocol. Extracted RNA was reverse transcribed to cDNA using a SuperScript III Reverse Transcriptase Kit (Thermo Fisher Scientific) prior to real-time quantitative PCR [RT-qPCR] analysis. RNA fragments encompassing the qPCR target were produced and used to generate an absolute standard curve as described previously.^33^ DENV genomic RNA was quantified using a RT-qPCR detection system (ABI) with a thermal profile of 95 °C for 3 min, 40 cycles of 95 °C for 2 s, and 60 °C for 20 s. The following primers were used for RT-qPCR analysis in this study:

DENV2 forward primer: 5′-TCG CTG CCC AAC ACA AG-3’.

DENV2 reverse primer: 5′-CAT GTT CTT TTT GCA TGT GAA C-3’.

### Mathematical modelling

**Supplementary Figure S1:**
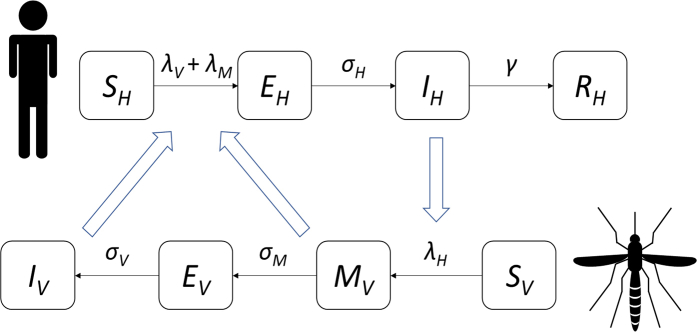
**Mathematical model of dengue transmission dynamics including a mechanical transmission component.** Once susceptible humans, S_H_, have been exposed to the virus, they become latently-infected with a force of infection comprised of a mechanical transmission component, λ_M_, and a viral amplification component, λ_V_. These latently-infected humans, E_H_, then become infectious after an average latency period of 1/ σ_H_. Infectious humans, I_H_, can recover after an average infectious period of 1/γ. Upon recovery, recovered humans, R_H_, are assumed to be immune to further infection events. Similarly, susceptible vectors, S_V_, become infected dependent on the force of infection due to infectious humans, λ_H_. They can then briefly transmit disease whilst in a mechanical transmission stage, M_V_, whose duration is given by 1/σ_M_. These females enter a latently-infected category, E_V_, where they remain for a duration equal to 1/σ_V_. After these females become infectious to humans, I_V_, they remain so for the duration of their lifespan.

**Supplementary Figure S2:**
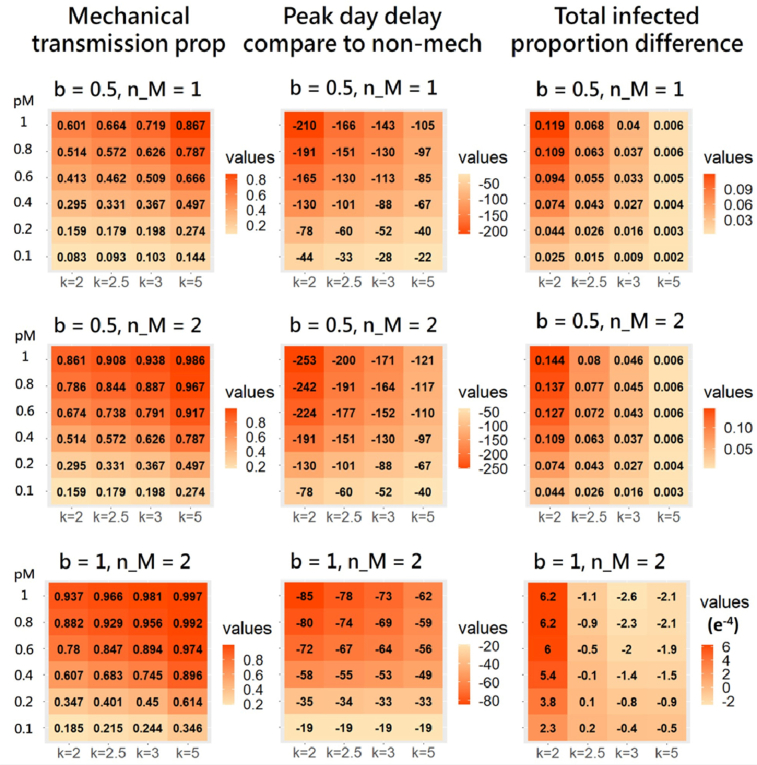
**The impact of mechanical transmission on population-level dengue infection dynamics under different parameter values.** Impact of altering key parameter values on dengue infection dynamics summarized by (left) the proportion of cases caused by MT, (centre) the change in the timing of peak incidence, and (right) the increase in the proportion of infected individuals after incorporating MT into the model. k = number of adult female mosquitoes per human; p_M_ = MT transmission probability; b = biting rate; n_M_ = number of bites during MT period.

A mathematical model of DENV transmission was created including both a susceptible-exposed-infectious-removed (SEIR) model for human transmission and a susceptible-mechanical transmitting-exposed-infectious (SMEI) model for mosquito transmission, where “M” represents the time period during which females could potentially mechanically transmit DENV following uptake of an infectious blood meal (Supplementary Figure S1). To achieve this, the vector transmission model of Esteva and Vargas^34^ was modified to enable more accurate determination of the relative contributions of MT and biological transmission following viral amplification in the mosquito midgut. With this model, the impact of MT was examined at different transmission settings by exploring a variety of parameter values that led to a wide range of estimates for the total proportion of the human population infected (Supplementary Table S1; Supplementary Table S2; see Methods section for selection of parameter ranges). Supplementary Figure S2 shows the impact of mechanical transmission on population-level dengue infection dynamics under different parameter values.

Humans are assumed to enter the population as susceptible, *S*_*H*_, and describe this population by

(1) $$\frac{{dS}_{H}}{dt}=\left(N_{H}-S_{H}\right)\mu_{H}\;-\;\left(\lambda_{V}+\lambda_{M}\right)S_{H}$$

here, *S*_*H*_ is the number of susceptible humans in the total population, *μ*_*H*_ is the birth rate (set as equal to the death rate), and *N*_*H*_ is the total human population size. The force of infection (the probability of an infection event per person per unit time) is split into two components, one part resulting from standard disease transmission following viral amplification in the mosquito, *λ*_*V*_, and a second part resulting from MT, *λ*_*M*_. The force of infection after viral amplification in the mosquito was estimated by

(2) $$\lambda_{V}=\frac{bp_{M}I_{V}}{N_{H}}\text{,}$$

where *I*_*V*_ is the number of DENV-infectious mosquitoes, *b* is the biting rate of mosquitoes, and *p*_*M*_ is the probability of mosquito-to-human transmission occurring when an infectious mosquito bites a susceptible human. Similarly, the force of infection resulting from MT was estimated by

(3) $$\lambda_{M}=\frac{bp_{M}M_{V}}{N_{H}}\text{,}$$

where *M*_*V*_ is the number of mosquitoes that have taken a blood meal and have the potential to transmit DENV mechanically, *b*_*M*_ is the biting rate of these mosquitoes, and *p*_*M*_ is the probability of mosquito-to-human transmission when a mosquito in this category bites a susceptible human.

Once susceptible humans have become infected, they progress to the latently-infected group (those that are infected but not yet infectious), *E*_*H*_, which is described by

(4) $$\frac{{dE}_{H}}{dt}=\left(\lambda_{V}+\lambda_{M}\right)S_{H}\;-\;\left(\sigma_{H}+\mu_{H}\right)E_{H}$$

Here, *E*_*H*_ is the number of latently infected humans in the total population, and *σ*_*H*_ is the progression rate by which an individual changes from being latently infected to being infectious. The population of infectious individuals, *I*_*H*_, is described by

(5) $$\frac{{dI}_{H}}{dt}=\sigma_{H}E_{H}\;-\;\left(\gamma +\mu_{H}\right)I_{H}\text{,}$$

where *γ* represents the rate at which an infectious individual recovers. These recovered individuals cannot be infected again, and the recovered population group is described by

(6) $$\frac{{dR}_{H}}{dt}=\gamma I_{H}\;-\;\mu_{H}R_{H}\text{,}$$

where *R*_*H*_ is the population of recovered humans. This model ignores other DENV-relevant factors, such as antibody-dependent enhancement and disease-induced mortality.

Mosquito population dynamics are modelled by a related set of equations. We assume that all female mosquitoes are born susceptible and that this susceptible population, *S*_*V*_, can be described by

(7) $$\frac{{ds}_{V}}{dt}=\left({kN}_{H}\left(1-\mathrm{acos}\left(2\pi t/T\right)\right)-S_{V}\right)\mu_{V}\;-\;\lambda_{H}S_{V}\text{.}$$

Here, *S*_*V*_ is the susceptible vector population, *k* is the average number of adult female mosquitoes per human, *μ*_*V*_ is the female mosquito mortality rate, *T* is the seasonal period (in this case, 1 year), and *a* is the degree of seasonality influencing the female mosquito population size (*a* = 0 in the absence of seasonality, and *a* = 1 if the peak mosquito emergence rate is twice the mean and the minimum emergence rate is 0). The force of infection, *λ*_*H*_, governing infection of mosquitoes by humans (when susceptible females bite infectious humans) is described by

(8) $$\lambda_{H}=\frac{bqI_{H}}{N_{H}}\text{,}$$

where *b* is the mosquito biting rate, and *q* is the probability of human-to-mosquito transmission. Before becoming latently infected, female mosquitoes pass through a MT stage, *M*_*V*_, described by

(9) $$\frac{{dM}_{V}}{dt}=\lambda_{H}S_{V}\;-\;\left(\sigma_{M}+\mu_{V}\right)M_{V}\text{,}$$

where *M*_*V*_ is the number of female mosquitoes in the MT group, and *σ*_*M*_ is the inverse of the duration of this phase. Following a latency period of duration *τ*_*V*_, mosquitoes enter an infectious stage (via standard transmission rather than MT), *I*_*V*_, described by

(10) $$\frac{{dI}_{V}}{dt}={\sigma_{M}M_{V}}_{\left(t\;-\;\tau_{V}\right)}\;\exp\left(-\mu_{V}\tau_{V}\right)\;-\;\mu_{V}I_{V}\text{,}$$

where *I*_*V*_ is the number of infectious females. A delay formulation was used to model the latently infected phase because the latent period is long relative to the lifetime of a mosquito (10 day latent period versus 14 day mean adult female lifespan^35^), hence an exponentially distributed time spent in the latently infected stage overestimates the number of vectors that are infectious following viral incubation within the mosquito. It was assumed that female mosquitoes cannot recover after infection, and thus remain infectious until death. The number of latently infected mosquitoes, *E*_*V*_, is described by

(11) $$\frac{{dE}_{V}}{dt}=\sigma_{M}M_{V}-{\sigma_{M}M_{V}}_{\left(t\;-\;\tau_{V}\right)}\;\exp\left(-\mu_{V}\tau_{V}\right)\;-\;\mu_{V}E_{V}\text{.}$$

Here, vertical transmission of dengue fever among vectors (from parent to offspring) was neglected.

Parameter estimates were sourced from previously published literature wherever possible (Supplementary Table S1), while MT parameters were based on results generated by this study. Select parameters were then explored in more depth via sensitivity analyses. The duration of MT, 1/*σ*_*M*_, was estimated at 1 h. The number of humans bitten during the MT period, *n*_*M*_, was also estimated; we assumed that a female mosquito would on average bite one human during the MT period immediately following her initial bite of an infectious human. The MT period biting rate was thus estimated to be

(12) $$b_{M}=n_{M}\sigma_{M}\text{,}$$

The probability of mosquito-to-human transmission for bites received by humans during the MT period was based on the experiments conducted on immunocompromised mice. Here, 16% and 40% of mice were infected after receiving two and four infective mosquito bites, respectively. Each mosquito bite was assumed to have an equal and independent probability of infection. This allowed for the probability of transmission after *k* bites, *p*_*k*_, to be described as

(13) $$p_{k}=1\;-\;{\left(1\;-\;p_{k}\right)}^{k}\text{,}$$

where *p*_*M*_ is the probability of transmission for each bite. Hence, *p*_*M*_ can be calculated as

(14) $$p_{M}=1\;-\;{\left(1\;-\;p_{k}\right)}^{\frac{1}{k}}\text{.}$$

Equation (14) yields a transmission probability of 0.12 per bite for the case of two infective bites, and of 0.083 per bite for the case of four infective bites, the mean of which is 0.10 per bite. This transmission probability was treated as an upper limit for all analyses, and a range of lower values (0.05, 0.025, 0.0125) was also considered in simulations to investigate potential differences between animal experiments and human transmission.

The initial parameters for each simulation were set as *I*_*H*_ = 10, *I*_*V*_ = 100, *N*_*H*_ = 10,000, and 0 for all other states. Furthermore, the following parameters were randomly generated from a range.

1. The average latent period in human host $\frac{1}{\sigma_{H}}$ is a randomly generated number from 3 to 5 days.
2. The average latent period in mosquito host $\tau_{V}=1/\sigma_{V}$ is a randomly generated number from 7 to 11 days.
3. The average infectious period in human host 1γ is a randomly generated number from 3 to 6 days.
4. The mosquito biting rate *b* is a randomly generated number from 0.5 to 2.
5. The number of bites during mechanical transmission period $n_{M}$ is randomly chosen from the set {1, 2, 3, 4} with equal probability.

The average number of adult female mosquitoes per person was set as 2. The average duration of MT was assumed to be 1 h, during which time a mosquito was expected to bite at least one other person (n_M_ = 1–4) with a probability of mechanical transmission per bite, *p*_*M*_, ranging from 100% to 10%. Model estimates were made based on household level infections i.e., individuals were infected within a household via bites from infected mosquitoes.

The basic reproduction number of infection, R_0_, for both conditions (with and without mechanical transmission) was estimated for a range of parameter values (Supplemental Tables S3–S6) by adapting formulae from previous papers.^36^, ^37^, ^38^ R_0_ estimates always increased following the addition of MT as a possible transmission route.

(15) $$R_{0}=\sqrt{\frac{1}{\mu_{v}^{2}N_{H}^{2}}\frac{{\left(b*p*I_{V}\;+\;b_{M}*p_{M}*M_{V}\right)}^{2}{\left(\sigma_{H}\;+\;\sigma_{V}\right)}^{2}{\left(q\right)}^{2}}{\left(\left(\sigma_{H}\;+\;\sigma_{V}\right)\;+\;\mu_{v}\right)\left(\left(\sigma_{H}\;+\;\sigma_{V}\right)\;+\;\mu_{H}\right)\left(\mu_{V}\;+\;\gamma \right)\mu_{H}}}$$

The code itself is available in a GitHub repository (https://github.com/roach231428/mechanical_transmission).

### Statistics

Mosquitoes were randomly selected from cages, and mice were assigned to different groups according to average weight. A significance level of *p* < 0.05 was used throughout (∗*p* < 0.05; ∗∗*p* < 0.01; ∗∗∗*p* < 0.001; ∗∗∗∗*p* < 0.0001). Mosquito and mouse serum titres were tested using non-parametric Mann–Whitney tests. Mouse body weights were tested using parametric unpaired t-tests. A log-rank (Mantel–Cox) test was used to compare survival distributions. Four biological replicates were conducted for all experiments. Statistical analyses were conducted using the GraphPad Prism 6 statistical software and R software.

### Role of funders

Study Funders had no role in study design, data collection, data analyses, interpretation, or writing.

## Results

### Mathematical models incorporating mechanical transmission suggest an influence on DENV transmission dynamics

**Fig. 2:**
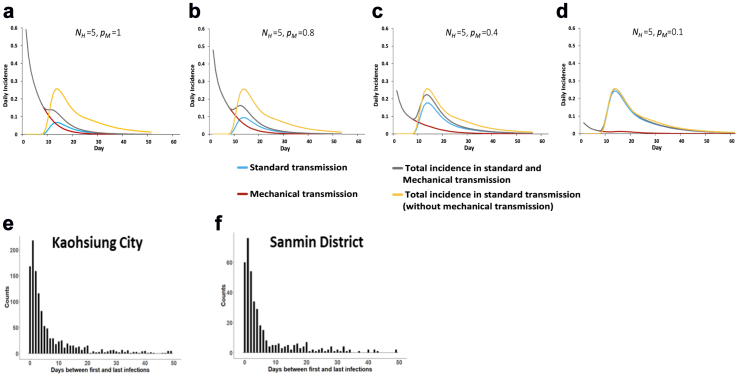
**Mechanical transmission of DENV may alter disease outbreak dynamics and influence the time between infections.** (**a and d)** Simulated daily incidence over the course of a dengue epidemic beginning with a single infected individual in an immunologically naïve population of 5 individuals (model in Equations (1), (2), (3), (4), (5), (6), (7), (8), (9), (10), (11), (12) in Supplementary Figure S1 and parameters in Supplementary Table S1). The average duration of MT is 1 h, during which time a mosquito is expected to bite at least one other person, n_M_ = 1∼2, with a probability of mechanical transmission per bite, *p*_*M*_, of **(a)** 100%, **(b)** 80%, **(c)** 40% or **(d)** 10%. The mosquito biting rate, b, was set to 0.5∼2, and the average number of adult female mosquitoes per person, k, was set to 2, 2.5, 3 or 5. The total daily incidence with MT (black line) or without MT (yellow line) is partitioned into incidence due to standard transmission following viral amplification in the vector (blue line) and incidence due to MT (red line). (**e and f)** Counts of cases throughout **(e)** all Kaohsiung City and **(f)** Sanmin district based on the number of days between first and last infections within a household during the 2015 Kaohsiung outbreak.

We first attempted to estimate MT's importance by creating a mathematical model comparing either pure biological transmission, or a mixture of the two transmission types. Fig. 2a–d shows the simulated daily incidence of DENV infections over the course of an epidemic for different probabilities of transmission per bite via MT, with a starting point of one infected individual per household of five individuals.

In all cases, provided *p*_*M*_ was greater than 0, cases of DENV occurred prior to the end of the necessary EIP. Fig. 2c acts as an illustration of our simulation results, with the probability of a new individual becoming infected in the same household on day 2 being greater than 84%, assuming an 80% probability of MT per bite. Furthermore, at least one of the five individuals became infected within three days. Details of the model and parameter ranges used in our simulations can be found in the Methods section and Supplementary Tables S1 and S2.

By studying dengue clusters from a 2015 outbreak not only within the entirety of Kaohsiung city, Taiwan but also specifically in significantly impacted district of Sanmin, we found a large number of cases reported within a household less than 3 days after the initial reported infection (Fig. 2e and f). Assuming a necessary EIP of at least several days, MT may act as one factor contributing towards this rapid spread of cases.

### Mechanical transmission of DENV is possible in mice models

**Supplementary Figure S3:**
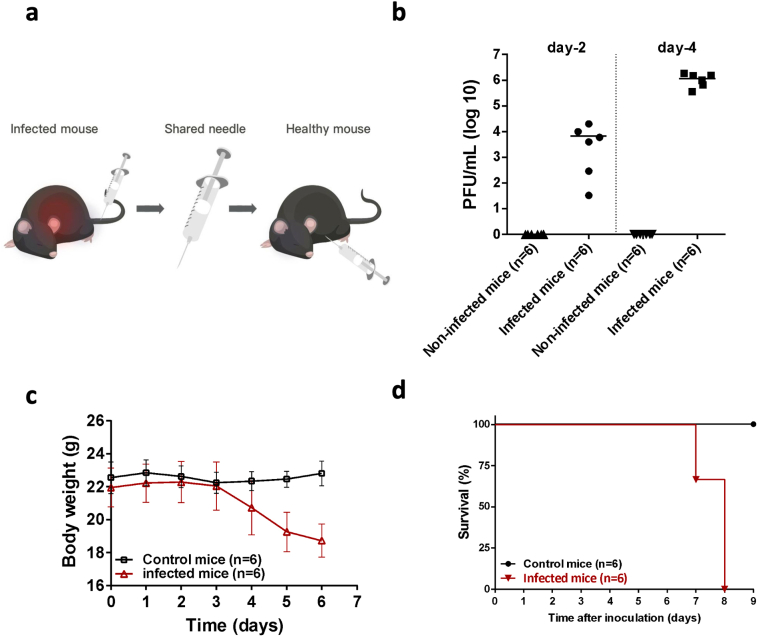
**Dengue transmitted by way of needle sharing causes viremia and mortality in mice.** (**a**) Schematic representation of DENV transmission via needle sharing between dengue-infected and naïve mice. (**b–d**) A naïve AGB6 mice intravenously injected with DENV (1000 PFU) at 6 dpi was used to create a DENV-contaminated needle for tail vein needle sticking. (**b**) Virus titre of DENV in serum, (**c**) body weight (**d**) and survival of male AGB6 which were intraperitoneally stuck four times with DENV-contaminated needles (n = 6 mice, infected) or sterile needles (n = 6, control). Data is presented as mean standard deviation for (**b and c**).

To investigate the potential of mechanical transmission (MT) of DENV, we conducted experiments using the cosmopolitan strain DENV-2 (TW2015) and immunocompromised AGB6 mice. These mice lack type I and type II interferon receptors,^39^ enabling robust replication of the virus in their spleens, lungs, and intestines.^19^ Utilising needle-stick assays, we tested whether DENV could be transferred via sharing of contaminated needles (Supplementary Figure S3a). Viremia, weight loss, and lethality occurred in mice that received the DENV-carrying needles, but not in those that received sterile needles, demonstrating the potential of MT of DENV (Supplementary Figure S3b–d).

**Fig. 3:**
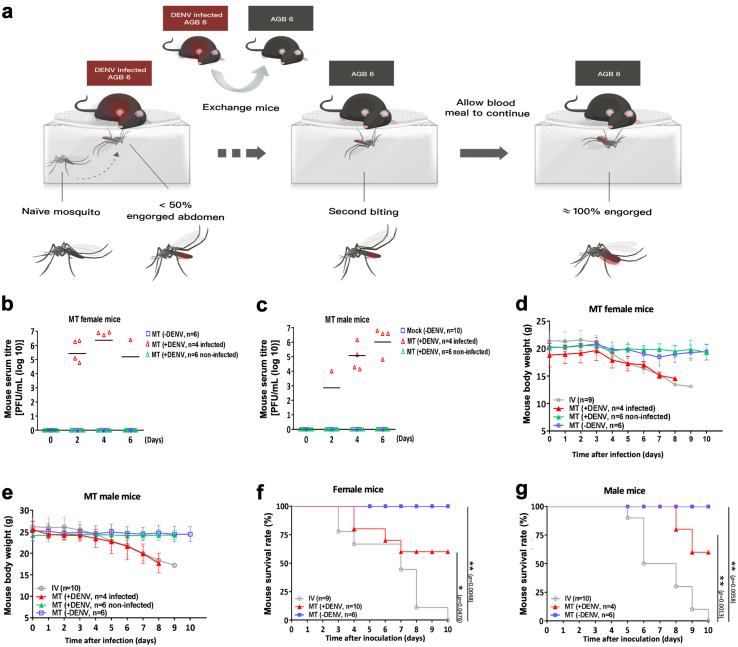
**Effects of the mechanical transmission of DENV to mice by female *A. aegyp*ti.** (**a)** Schematic of animal model created to investigate mechanical transmission of DENV by *A. aegypti* mosquitoes. *A. aegypti* mosquitoes at most 50% engorged with blood were used as a vector for DENV transmission via mechanical biting without biological DENV replication. *Step one*: AGB6 mice (red) were intravenously infected with DENV. *Step two*: Mosquitoes were allowed to feed on these mice until at most half engorged, after which mice were switched with naïve AGB6 mice (grey). *Step three*: Mosquitoes fed on these naïve AGB6 mice (referred to as MT mice), infecting them via mechanical transmission. **(b**–**g)** Serum DENV titre evaluated by plaque-formation assay **(b, c)**, body weight **(d, e)** and survival **(f, g)** were examined in female (n = 10) and male (n = 10) mice infected with DENV or in mice (n = 6 for male or female individually) without DENV infection via mechanical transmission (MT) by four bites of DENV-exposed *A. aegypti* or *A. aegypti* alone or in mice (n = 9 for female or n = 10 for male) infected with DENV with intravenous injection. Kaplan–Meier survival curves are shown in **(f, g)**. Data is presented as mean standard deviation for **(b**–**e)**. The number of mice used in each group per individual experiment are highlighted. ∗*p* < 0.05; ∗∗*p* < 0.01. Details of statistical analysis are provided in the Methods.

We then moved on to investigate the possibility of transmission through the female proboscis of *Ae. aegypti* mosquitoes. We starved individual female mosquitoes for 24 h and then allowed them to bite infected mice which had been injected with DENV three days prior. After partial completion of a blood meal, infected mice were replaced with naïve mice, which were bitten by female mosquitoes to complete their blood meals (Fig. 3a).

As for the needlestick experiment, naïve male and female mice bitten by infected mosquitoes could become infected (40% infectivity rate) as evidenced by detectable DENV serum titers (Fig. 3b and c). Successful transmission of DENV was associated with a loss of body weight,^19^^,^^40^ as seen in mice that were either infected through MT or through direct tail vein inoculation (Fig. 3d and e). Infected mice also died (Fig. 3f and g), confirming the existence of MT as a lethal method of transmission for DENV. MT of DENV is thus possible by naïve mosquitoes immediately following incomplete biting of infected hosts, with this transmission sufficiently significant as to induce mortality.

### Residual DENV titres in the mosquito proboscis act as a determinant for mechanical transmission success

Although HIV transmission via contaminated needles is well-known,^41^ the minimum infectious dose remains unclear. Therefore, while we hypothesized that DENV in the mosquito proboscis could be responsible for infecting naïve mice, a sufficient concentration of the virus should be required for an infection to occur. Indeed, naïve AGB6 mice bitten by fewer than four half engorged female mosquitoes did not show obvious symptoms of DENV infection (such as lethality, body weight loss, and serum DENV titre; Supplementary Figure S2a–d). Hence, we aimed to confirm the presence of DENV in the partially fed mosquito proboscis and investigate the minimum titres required for infection.

**Fig. 4:**
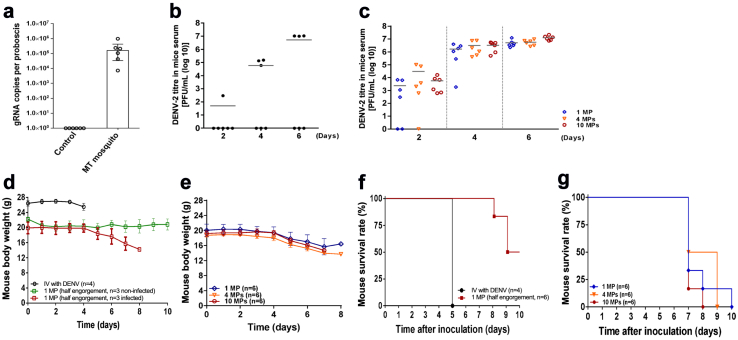
**Residual DENV in proboscis of *A. aegypti* mediates dengue spread via mechanical transmission.** (**a)** Copies of dengue genomic RNA in the proboscis of a mosquito after biting DENV-infected or non-infected mice. Mosquito proboscises (n = 10) were collected and examined for each experiment/group. Six independent experiments were performed. (**b)** Virus titre of DENV-2 in mice serum, **(d)** body weight, and **(f)** Kaplan–Meier survival curves of male mice intravenously injected with DENV **(d, f)** (n = 4 mice) or the extracts from one half engorged mosquito proboscis (MP) from DENV blood-exposed *A. aegypti* mosquitoes **(b, d, and f)** (n = 6 mice per group for proboscis extract injection; n = 3 for male mice infected with DENV via MT; n = 3 for male mice bitten but not infected with DENV via MT). (**c)** Virus titre of DENV-2 in mice serum, **(e)** body weight, and **(g)** Kaplan–Meier survival curves of male mice intravenously injected with the extracts from either 1, 4 or 10 fully engorged mosquito proboscises (MPs) from DENV blood-exposed *A. aegypti* mosquitoes (n = 6 mice per group for injection of proboscis extracts). The number of mice used in each group per individual experiment is highlighted. Data is presented as mean standard deviation for **(b**–**e)**. 1 MP, extracts from one mosquito proboscis injected; 4 MP, extracts from four mosquito proboscises injected; 10 MP, extracts from ten mosquito proboscises injected.

Initially, using RT-qPCR, we identified DENV genomic RNA in the proboscis of females after a single incomplete bite from DENV-infected mice (estimated 10^5^ copies) (Fig. 4a). Subsequently, we aimed to correlate the detrimental effects resulting from MT with the residual DENV titre found within the mosquito proboscis. To achieve this, we dissected proboscis samples from *Ae. aegypti* that had obtained incomplete or complete blood meals from biting DENV-infected mice. These proboscis extracts were tail-vein injected into AGB6 naïve mice to test DENV virulence. Notably, we detected infectious DENV in the serum after intravenous injection (Fig. 4b and c), which was associated with body weight loss (Fig. 4d and e) and lethality (Fig. 4f and g).

### Mechanical transmission contributes to the speed and intensity of DENV outbreaks in mathematical models

To investigate the potential proportion of DENV infections resulting from MT in human populations we developed a susceptible-exposed-infectious-removed (SEIR) mathematical model of single-strain dengue transmission^42^ incorporating a MT component^34^ (Supplementary Figure S1) to study population-level dengue infection dynamics. The total number of people infected increased by around 2.5%, with approximately 8.3% of these resulting directly from MT, when we set the average number of adult female mosquitoes per person [*k*] = 2, mosquito biting rate [*b*] = 0.5, MT probability [*p*_*M*_] = 0.1, number of bites [*n*_*M*_] = 1. When the MT probability [*p*_*M*_] increases from 0.1 to 1, the total number people infected increased by around 12%, with approximately 60% of these resulting directly from MT (Supplementary Table S2; Supplementary Figure S2).

**Fig. 5:**
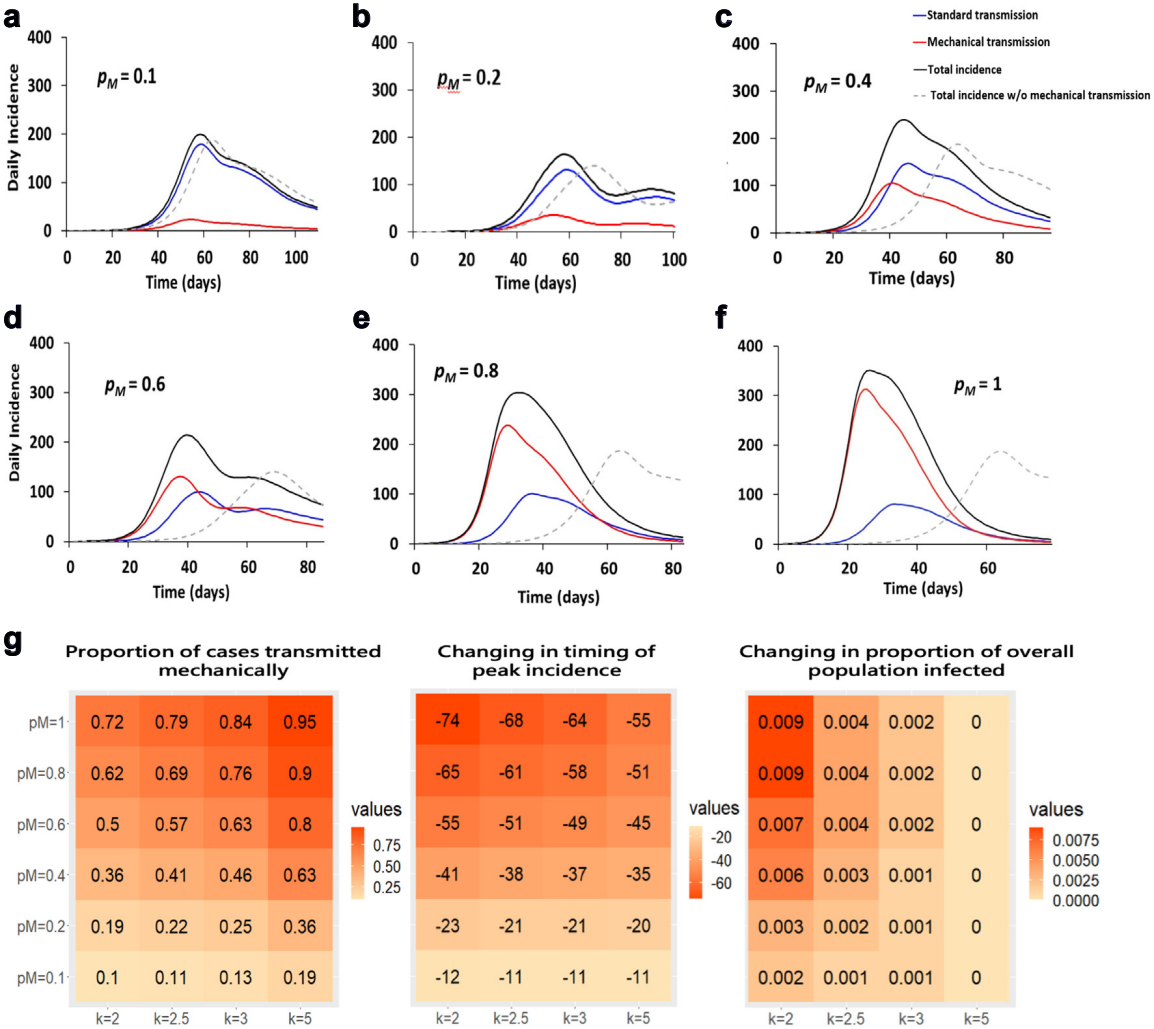
**Mathematical model of dengue dynamics incorporating mechanical transmission.** (**a-f)** Simulated daily incidence over the course of a dengue epidemic beginning with a single infected individual in an immunologically naïve population of 10,000 individuals (model in Equations (1), (2), (3), (4), (5), (6), (7), (8), (9), (10), (11), (12) in Supplementary Figure S3 and parameters in Supplementary Table S1). The average duration of MT is 1 h, during which time a mosquito is expected to bite one to four other person(s), n_M_ = 1∼4, with a probability of mechanical transmission per bite, p_M_, of **(a)** 10%, **(b)** 20%, **(c)** 40%, **(d)** 60%, **(e)** 80%, or **(f)** 100%. The mosquito biting rate, b, was set to be a randomly generated number from 0.5 to 2, and the average number of adult female mosquitoes per person, k, was set to 2.5. The total daily incidence (black line) is partitioned into incidence due to standard transmission following viral amplification in the vector (blue line) and incidence due to MT (red line). For comparison, the incidence when MT is not allowed (*p*_*M*_ = 0) is shown by the grey dashed line. **(g)** The impact of MT on dengue infection dynamics is summarized by the proportion of cases caused by MT, the change in the timing of peak incidence, and the increase in the proportion of infected individuals after incorporating MT into the model under a range of *p*_*M*_ and k values.

Modifying the biting rate *b* to be randomly generated from 0.5 to 2 and setting MT probability *p*_*M*_ = 0.1, 0.2, 0.4, 0.6, 0.8, 1 (Supplementary Table S2), we explored a wide range of estimates for the total proportion of the human population infected (Fig. 5a–g). In this case, with *p*_*M*_ = 0.1, almost 11% of infected individuals were infected via MT (Fig. 5a and g). For a higher MT probability, *p*_*M*_ = 0.8, the total number of infected individuals increased by 15% compared to the total number of infected individuals for *p*_*M*_ = 0.1, 69% of whom were infected directly from MT (Fig. 5e and g). For the highest MT probability, *p*_*M*_ = 1 (the experimental value), the total number of infected individuals increased by 16.5% compared to the total number of infected individuals for *p*_*M*_ = 0.1, 79% of whom were infected directly from MT (Fig. 5f). The change in the time of peak incidence also varied considerably, with *p*_*M*_ = 0.1 resulting in a 11 day forward shift as compared to a model without mechanical transmission (Fig. 5g).

Depending on the parameters used, this change in peak incidence could range from a minimum of 11 days–74 days earlier than for models where transmission follows only viral amplification (Fig. 5g; Supplementary Figure S2). The magnitude of this peak appeared increased for models containing MT; in the outbreaks modelled in Fig. 5, daily incidence can peak at around 15% greater than for the model without MT.

The total number of infected individuals also increased by between 0–14.4% and 8.3–99.7% of dengue cases were estimated to be acquired via MT over the course of a simulated epidemic (Fig. 5; Supplementary Figure S2). Thus, for a wide range of parameter values, MT is expected to influence not only the timing, but also the severity, of DENV outbreaks.

We then explored how the proportion of cases acquired through MT over the course of the epidemic changes with the number of bites during the MT period, *n*_*M*_, and the probability of mosquito-to-human transmission during MT, *p*_*M*_ (Supplementary Figure S2). Even for a low MT probability, the proportion of cases transmitted by this route was non-negligible; for *p*_*M*_ = 0.1 with *b* = 0.5, the proportion of MT cases was predicted to be 8.3–14.4% for *n*_*M*_ = 1 and 15.9–27.4% for *n*_*M*_ = 2. For the highest MT probability of 100% per bite, the proportion of cases resulting from MT rises to 60.1–86.7% for *n*_*M*_ = 1 and 86.1–98.6% for *n*_*M*_ = 2.

Taken in conjunction with the results of our DENV infection experiments, these modelling results indicate that MT of DENV is possible by *Ae. aegypti* that engage in multiple biting events. While the extent of MT in human populations is unknown, it is conceivable that it makes a non-negligible contribution to DENV transmission dynamics, especially in densely populated regions.

## Discussion

In this study, we investigated the transmission routes of dengue virus in *Ae. aegypti* mosquitoes. Previous studies have shown that these mosquitoes can transmit DENV biologically, but the possibility of mechanical transmission has not been fully explored. Our findings show that *Ae. aegypti*-mediated DENV infection can be transmitted both biologically and mechanically. Our study used mice-based transmission assays to show that around 25% of mice can become infected with DENV from mosquitoes that had previously bitten infectious mice, similar to other bloodborne viruses like hepatitis B virus (HBV).^43^^,^^44^ Mathematical modelling further suggests that MT may account for a significant percentage of overall transmission and shift the timing of peak disease incidence forward. MT may significantly impact the spread of dengue outbreaks, especially in households or other settings where mosquitoes have access to multiple susceptible hosts. Such modelling of MT could be incorporated into existing models testing the potential effects of mosquito control/interventions on DENV disease dynamics.^37^

Our analyses suggest that targeting potential DENV-relevant receptors in mosquitoes may offer a control option for reducing the risk of dengue transmission. Several putative receptors or attachment factors in *A. aegypti* or mosquito cells, including prohibitin, protein R67, R80, and glycoprotein family members such as gp40 and gp45, have been associated with DENV recognition.^45^ Further research is needed to determine the minimum viral loads required for MT to occur and to understand the mechanisms involved in the interactions between DENV and the mosquito proboscis. Our study strongly supports MT as playing a role in dengue transmission via DENV-contaminated proboscises.

Recent work demonstrating the increased attractiveness of mice infected with flaviviruses to host seeking *Aedes* females may also be relevant from a MT perspective, as this could increase the likelihood of female mosquitoes first biting those infected before completing their blood meal (and potentially transmitting disease) by biting non-infected individuals.^46^ This suggests individuals within the same household are potentially not equally likely to be bitten, supporting an increased speed of disease transmission. With our current experimental setup, we were unable to exclude the possibility of the influence of time intervals between bites by infected mosquitoes on MT efficiency, resulting in potential changes in load or longevity of residual DENV.

There are several limitations to this study that need to be addressed in future experiments. For example, the use of immunocompromised mice limits the generalization of our results to the human population. In addition, the influence of time intervals between bites by infected mosquitoes on MT efficiency, and the residual load or longevity of DENV, remains unclear. Despite these limitations, our results have important implications for the control of dengue outbreaks and highlight the need for further research into the role of MT in dengue transmission.

## Contributors

HHL, SCW and HWW conducted experiments.

HHL, SCW, KNT and HWW worked on the spatial distribution of mechanical transmission during dengue epidemics.

PJC, WTT and HWC prepared the materials for dengue virus infection and mice.

HHL, MPS, SCW, HHT, MCC, YCC, CYP, KNT, GHC, YAY, CKT, HHC, LRH and CHC participated in the experimental design and data interpretation/analysis.

HHT, MCC, YCC, HHC, MPS and JMM conducted the mathematical modelling.

HHL, MPS, SCW, JMM, KNT, OSA, GYY and CHC prepared the manuscript.

All authors read and approved the final version of the manuscript. HHL, SCW and CHC verified the underlying data used for all analyses.

## Data sharing statement

All experimental data collected is available via Dryad: https://doi.org/10.5061/dryad.1vhhmgqzb.

All mathematical modelling scripts are available via GitHub: https://github.com/roach231428/mechanical_transmission.

## Declaration of interests

OSA. is a founder of Agragene, Inc. with equity interest and a founder of Synvect with equity interest. The terms of this arrangement have been reviewed and approved by the University of California, San Diego in accordance with its conflict of interest policies. All remaining authors declare no competing interests.

## Figures and Tables

**Supplementary Figure S4:**
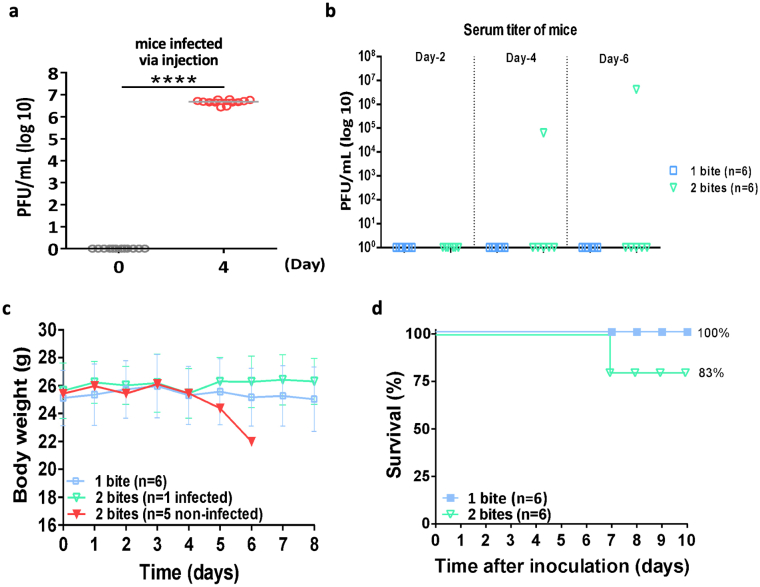
**Effects on AGB6 mice caused by mechanical transmission of dengue by *A aegypti*. (a)** DENV titre in the serum of female and male mice was examined by plaque-formation assay at four days after intravenous infection with DENV. Circles represent means of virus titre from DENV-inoculated AGB6 mice (n = 12). **(b–d) (b)** Serum DENV, **(c)** body weight **(d)** and survival were examined in male mice (n = 6 for each group) mice potentially infected with DENV via mechanical transmission by one or two bites of DENV exposed *A. aegypti*. Data is presented as mean standard deviation for **(a and c)**. ∗∗∗∗*p*<0.001. Details of statistical analysis are provided in the Methods.
