# Supplementary material for: Mechanical transmission of dengue virus by Aedes aegypti may influence disease transmission dynamics during outbreaks
Source: eBioMedicine. 2023 Jul 22;94:104723. doi: 10.1016/j.ebiom.2023.104723 (PMC10382859; doi:10.1016/j.ebiom.2023.104723)
Supplement: Supplementary Tables [file mmc1.docx]

**Table S1. Table of parameter estimates used in the dengue transmission model.**

| **Parameter:** | **Description:** | **Value** | **Reference:** |
| --- | --- | --- | --- |
| _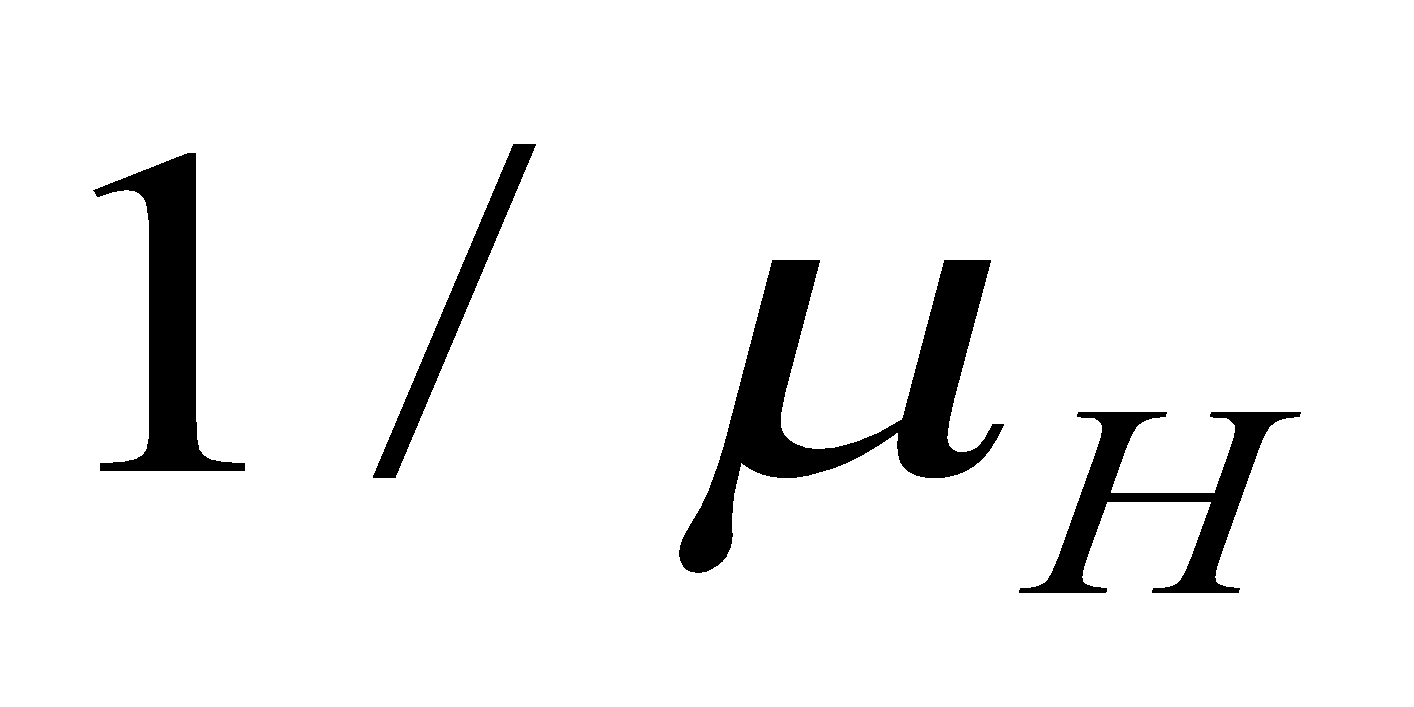_ | Average adult human lifetime | 70 years |  |
| *N_H_* | Human population size | 10,000 |  |
| *I_H_* | The population of infectious individuals | 10 |  |
| *I_V_* | the number of infectious female mosquito | 100 |  |
| *k* | Average number of adult female mosquitoes per person | 2, 2.5, 3, 5 | [(1,2)](https://paperpile.com/c/Zw2KIw/iSPj+wopb) |
| _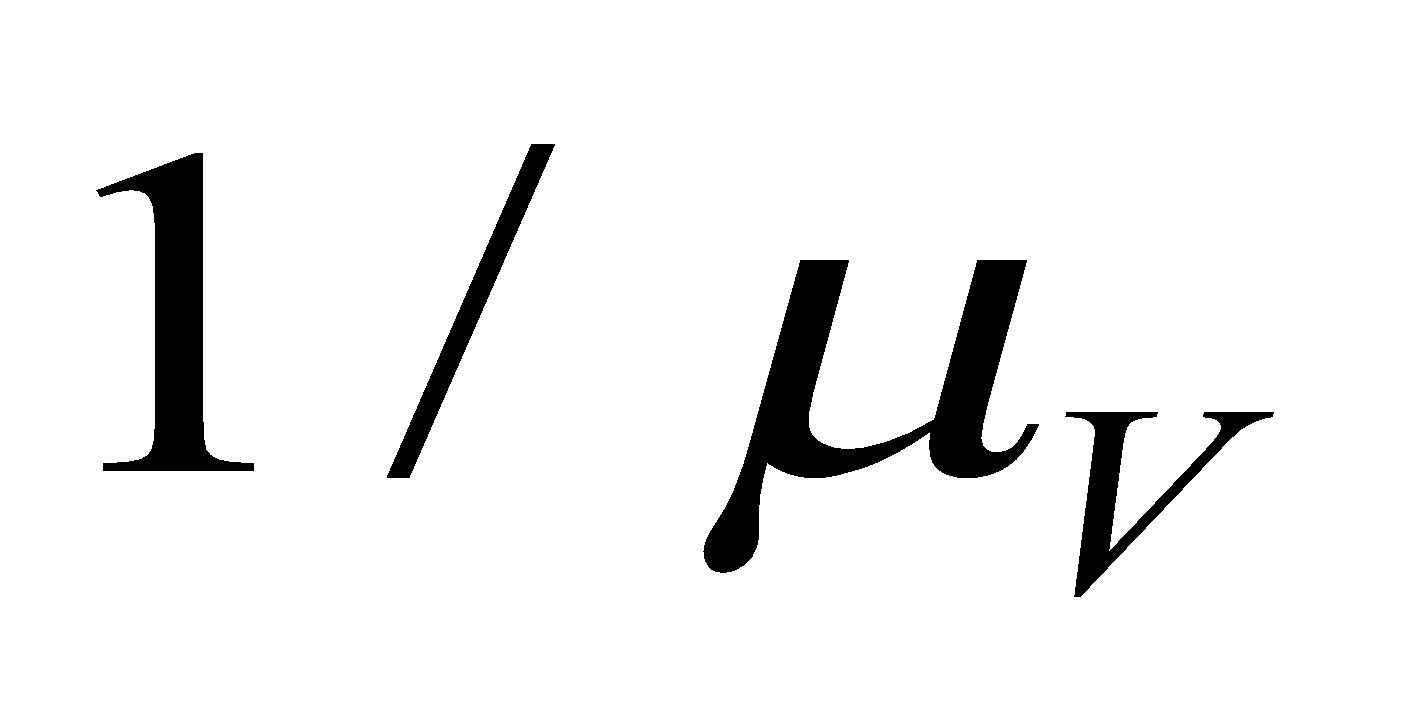_ | Average adult female mosquito lifetime | 14 days | [(3–5)](https://paperpile.com/c/Zw2KIw/6tdy+hU58+uyiP) |
| _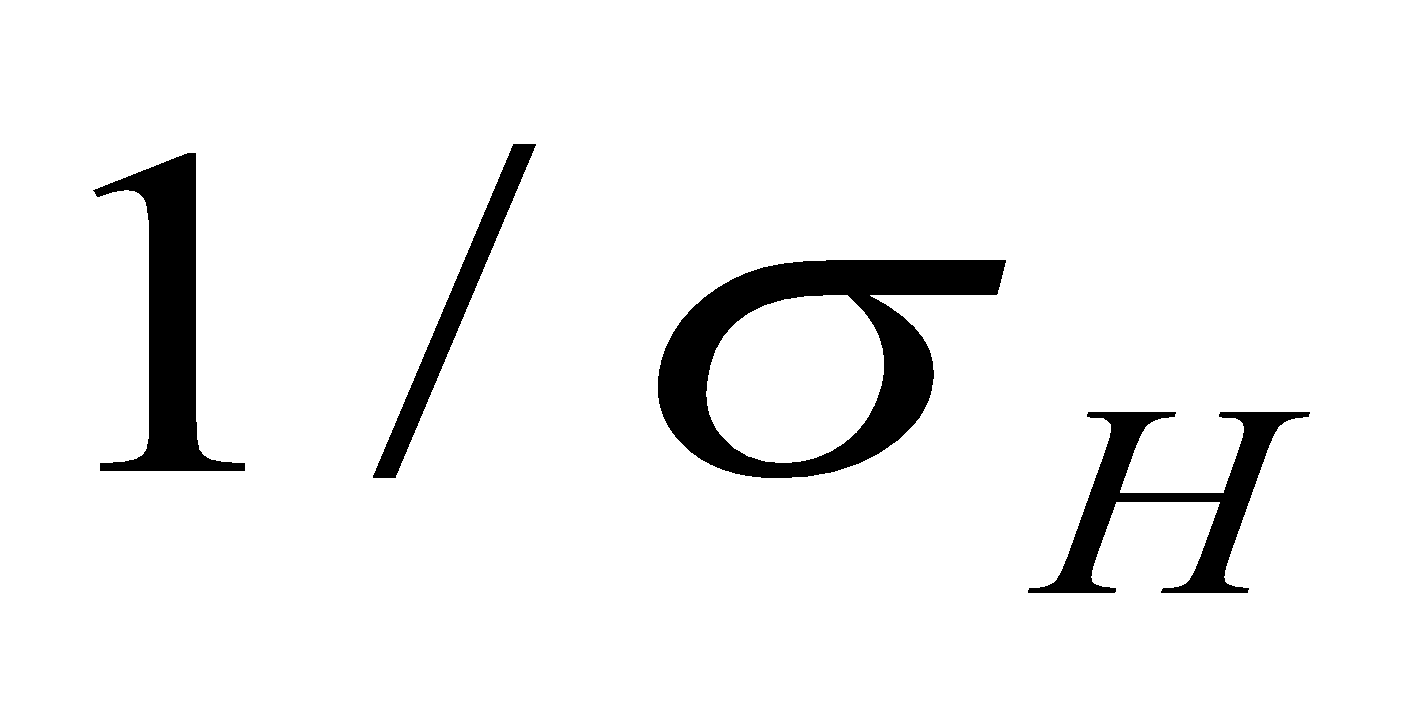_ | Average latent period in human host | a randomly generated number from 3 to 5 days | [(6,7)](https://paperpile.com/c/Zw2KIw/CZEs+DyiY) |
| _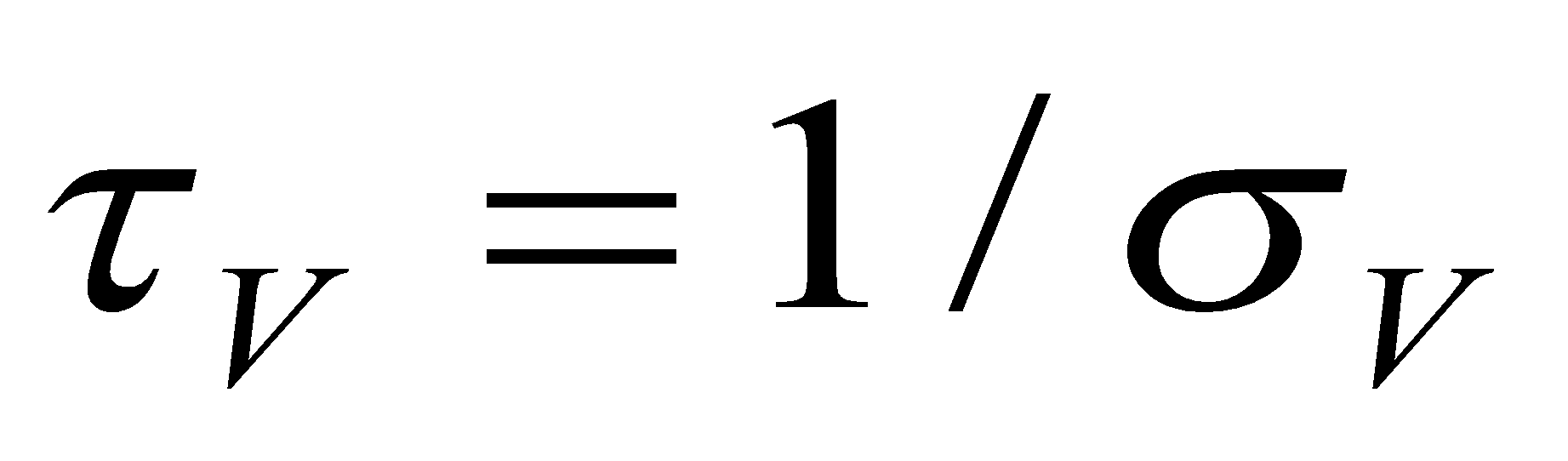_ | Average latent period in mosquito host | a randomly generated number from 7 to 11 days | [(6,8)](https://paperpile.com/c/Zw2KIw/CZEs+4aS3)  (Watts et al., 1987) |
| _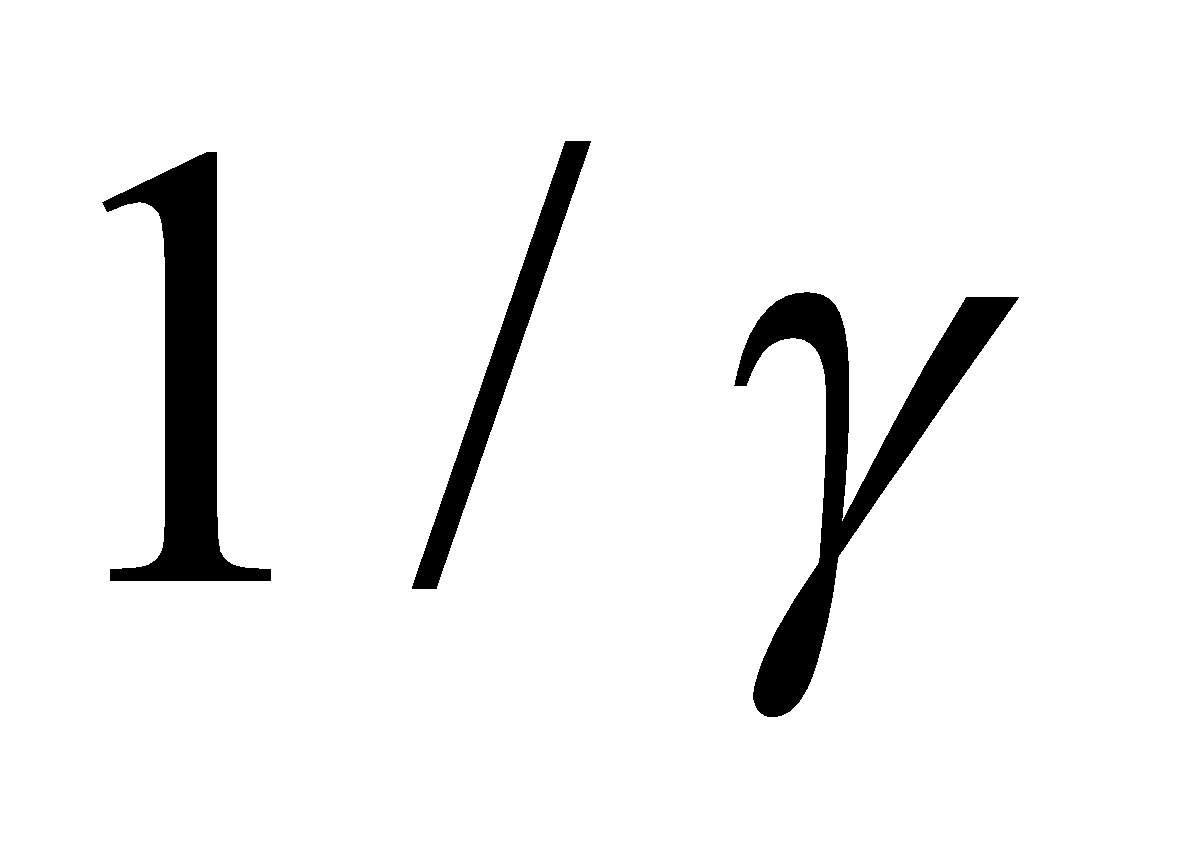_ | Average infectious period in human host | a randomly generated number from 3 to 6 days | [(9,10)](https://paperpile.com/c/Zw2KIw/jTEf+P4nF) |
| *b* | Mosquito biting rate | a randomly generated number from 0.5 to 2 | [(4,11)](https://paperpile.com/c/Zw2KIw/hU58+DxuO) |
| *p* | Mosquito-to-human transmission probability (following parasite incubation) | 0.38 | [(12)](https://paperpile.com/c/Zw2KIw/m6DH) |
| *q* | Human-to-mosquito transmission probability | 0.38 | [(12)](https://paperpile.com/c/Zw2KIw/m6DH) |
| *a* | Degree of seasonality in mosquito population size | 0 |  |
| *T* | Period of seasonality | 1 year |  |
| _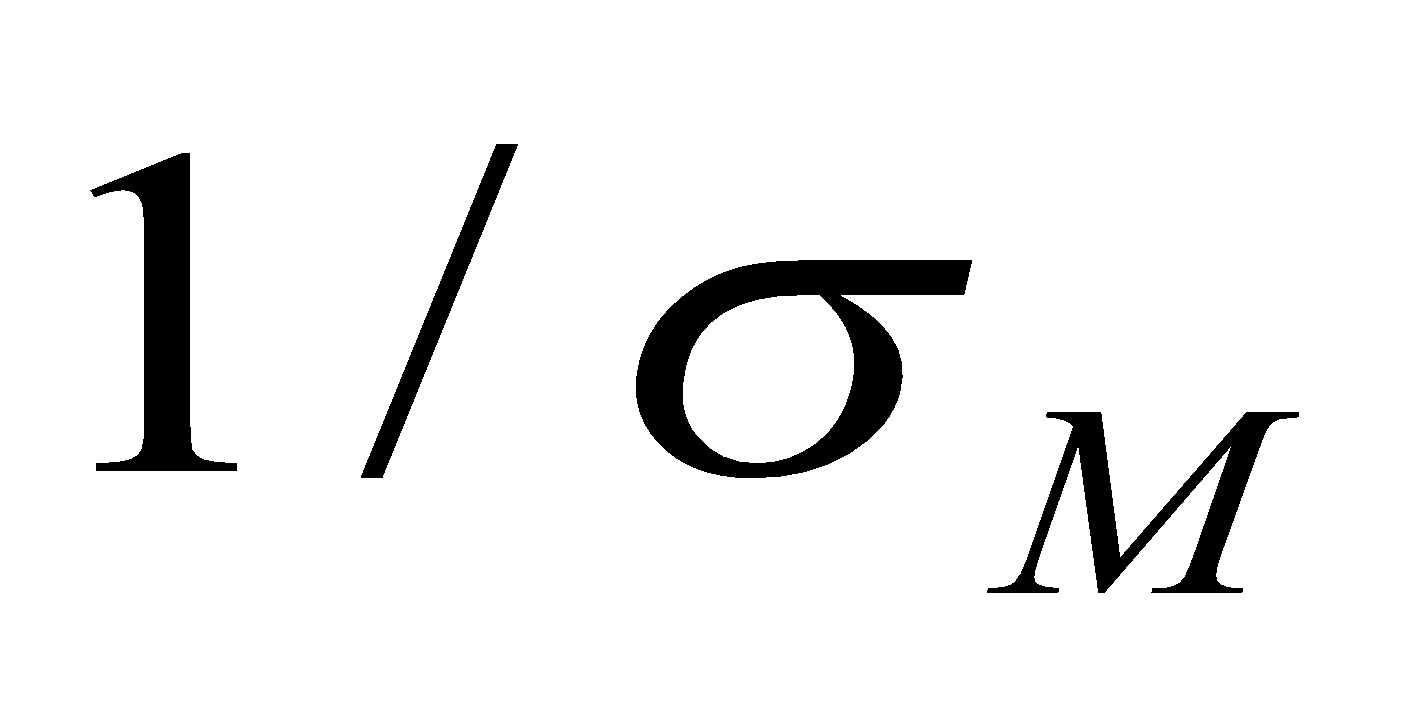_ | Average duration of mechanical transmission | 1 hour |  |
| *n_M_* | Number of bites during mechanical transmission period | randomly chosen from the set {1, 2, 3, 4} with equal probability |  |
| *p_M_* | Mosquito-to-human transmission probability during mechanical transmission | 0.1, 0.2, 0.4, 0.6, 0.8, 1 |  |

**Table S2. Changes in expected proportion of human population infected for a range of model parameters.**

| (*b*, *n_M_*)  *k* | (0.3, 1) | (0.5, 1) | (0.3, 2) | (0.5, 2) |
| --- | --- | --- | --- | --- |
| 2 | 11.27%* | 91.36% | 11.27% | 91.36% |
| 2.5 | 43.67% | 95.54% | 43.67% | 91.54% |
| 3 | 62.05% | 97.56% | 62.05% | 97.56% |
| 5 | 89.36% | 99.65% | 89.36% | 99.65% |

*Similar to the 2015 outbreak in Kaohsiung.

**Table S3. Changes in basic reproduction number for model parameters Iv=100, Mv=2**

|  | *p_M_*=0 | *p_M_*=0.1 | *p_M_*=0.4 | *p_M_*=0.8 | *p_M_*=1 |
| --- | --- | --- | --- | --- | --- |
| mean | 6.9212 | 8.858 | 14.6683 | 22.4153 | 26.2889 |
| median | 6.215 | 8.2961 | 14.4652 | 22.4579 | 26.5383 |
| 95% CI (Lower) | 6.112 | 7.9696 | 13.3016 | 20.2061 | 23.6313 |
| 95% CI (Upper) | 7.7304 | 9.7464 | 16.0349 | 24.6246 | 28.9464 |

**Table S4. Changes in basic reproduction number for model parameters Iv=100, Mv=1**

|  | *p_M_*=0 | *p_M_*=0.1 | *p_M_*=0.4 | *p_M_*=0.8 | *p_M_*=1 |
| --- | --- | --- | --- | --- | --- |
| mean | 6.9212 | 7.8896 | 10.7947 | 14.6683 | 16.605 |
| median | 6.215 | 7.1386 | 10.2751 | 14.4652 | 16.401 |
| 95% CI (Lower) | 6.112 | 7.0482 | 9.7768 | 13.3016 | 15.0391 |
| 95% CI (Upper) | 7.7304 | 8.731 | 11.8127 | 16.0349 | 18.171 |

**Table S5. Changes in basic reproduction number for model parameters Iv=10, Mv=2**

|  | *p_M_*=0 | *p_M_*=0.1 | *p_M_*=0.4 | *p_M_*=0.8 | *p_M_*=1 |
| --- | --- | --- | --- | --- | --- |
| mean | 0.6921 | 2.6289 | 8.4392 | 16.1863 | 20.0598 |
| median | 0.6215 | 2.6538 | 8.7214 | 16.7197 | 20.7153 |
| 95% CI (Lower) | 0.6112 | 2.3631 | 7.4707 | 14.2706 | 17.6701 |
| 95% CI (Upper) | 0.773 | 2.8946 | 9.4077 | 18.1019 | 22.4494 |

**Table S6. Changes in basic reproduction number for model parameters Iv=10, Mv=1**

|  | *p_M_*=0 | *p_M_*=0.1 | *p_M_*=0.4 | *p_M_*=0.8 | *p_M_*=1 |
| --- | --- | --- | --- | --- | --- |
| mean | 0.6921 | 1.6605 | 4.5657 | 8.4392 | 10.376 |
| median | 0.6215 | 1.6401 | 4.7016 | 8.7214 | 10.7144 |
| 95% CI (Lower) | 0.6112 | 1.5039 | 4.0684 | 7.4707 | 9.1709 |
| 95% CI (Upper) | 0.773 | 1.8171 | 5.0629 | 9.4077 | 11.581 |

**SI References**

1. [Newton EA, Reiter P. A model of the transmission of dengue fever with an evaluation of the impact of ultra-low volume (ULV) insecticide applications on dengue epidemics. Am J Trop Med Hyg. 1992 Dec;47(6):709–20.](http://paperpile.com/b/Zw2KIw/iSPj)

2. [Focks DA, Brenner RJ, Hayes J, Daniels E. Transmission thresholds for dengue in terms of Aedes aegypti pupae per person with discussion of their utility in source reduction efforts. Am J Trop Med Hyg. 2000 Jan;62(1):11–8.](http://paperpile.com/b/Zw2KIw/wopb)

3. [Sheppard PM, Macdonald WW, Tonn RJ, Grab B. The dynamics of an adult population of Aedes aegypti in relation to dengue haemorrhagic fever in Bangkok. J Anim Ecol. 1969 Oct;38(3):661.](http://paperpile.com/b/Zw2KIw/6tdy)

4. [Trpis M, Hausermann W. Dispersal and other population parameters of Aedes aegypti in an African village and their possible significance in epidemiology of vector-borne diseases. Am J Trop Med Hyg. 1986 Nov;35(6):1263–79.](http://paperpile.com/b/Zw2KIw/hU58)

5. [Trpis M, Häusermann W, Craig GB Jr. Estimates of population size, dispersal, and longevity of domestic Aedes aegypti aegypti (Diptera: Culicidae) by mark-release-recapture in the village of Shauri Moyo in eastern Kenya. J Med Entomol. 1995 Jan;32(1):27–33.](http://paperpile.com/b/Zw2KIw/uyiP)

6. [Siler JF, Hall MW, Hitchens AP. Dengue: Its History, Epidemiology, Mechanism of Transmission, Etiology, Clinical Manifestations, Immunity, and Prevention. By J.F. Siler, Milton W. Hall, and A. Parker Hitchens. 1926.](http://paperpile.com/b/Zw2KIw/CZEs)

7. [Sabin AB. Research on dengue during World War II. Am J Trop Med Hyg. 1952 Jan;1(1):30–50.](http://paperpile.com/b/Zw2KIw/DyiY)

8. [Watts DM, Burke DS, Harrison BA, Whitmire RE, Nisalak A. Effect of temperature on the vector efficiency of Aedes aegypti for dengue 2 virus. Am J Trop Med Hyg. 1987 Jan;36(1):143–52.](http://paperpile.com/b/Zw2KIw/4aS3)

9. [Gubler DJ. Dengue and dengue hemorrhagic fever. Clin Microbiol Rev. 1998 Jul;11(3):480–96.](http://paperpile.com/b/Zw2KIw/jTEf)

10. [Vaughn DW, Green S, Kalayanarooj S, Innis BL, Nimmannitya S, Suntayakorn S, et al. Dengue viremia titer, antibody response pattern, and virus serotype correlate with disease severity. J Infect Dis. 2000 Jan;181(1):2–9.](http://paperpile.com/b/Zw2KIw/P4nF)

11. [Putnam JL, Scott TW. Blood-feeding behavior of dengue-2 virus-infected Aedes aegypti. Am J Trop Med Hyg. 1995 Mar;52(3):225–7.](http://paperpile.com/b/Zw2KIw/DxuO)

12. [Wearing HJ, Rohani P. Ecological and immunological determinants of dengue epidemics. Proc Natl Acad Sci U S A. 2006 Aug 1;103(31):11802–7.](http://paperpile.com/b/Zw2KIw/m6DH)
